# Chitin-Methacrylate: Preparation, Characterization and Hydrogel Formation

**DOI:** 10.3390/ma4101728

**Published:** 2011-10-06

**Authors:** Eugene Khor, Hong Wu, Lee Yong Lim, Chang Ming Guo

**Affiliations:** 1 Department of Chemistry, the National University of Singapore, 3 Science Drive 3, Singapore 117543, Singapore; E-Mail: wuhongsg@gmail.com; 2 Pharmacy, School of Biomedical, Biomolecular and Chemical Sciences, The University of Western Australia, 35 Stirling Highway, Crawley WA 6009, Australia; E-Mail: limly@cyllene.uwa.edu.au; 3 Department of Orthopedic Surgery, Singapore General Hospital, Outram Road, Singapore 16960, Singapore; E-Mail: guo.chang.ming@sgh.com.sg

**Keywords:** chitin-methacrylate, chitin derivatization, hydrogel, degradation, cytotoxicity

## Abstract

Chitin-methacrylate (CM) was prepared by the reaction of methacrylic acid on chitin in 5% LiCl/DMAc in the presence of N,N’-dicyclocarbodiimide and dimethylaminopyridine. The resultant chitin-methacrylate product was isolated in 61% yield and was found to be readily water-soluble. The derivative was found to be a mixture of methacrylate and methacrylic-dimethylaminopyridine complex substituents at the C-6 position in approximately equal amounts. In order to evaluate the activity of the methacrylate double bond, a chitin-methacrylate water solution was photo-crosslinked in the presence of Irgacure 2959 photo-initiator to generate CM-hydrogel. The CM-hydrogel was evaluated for its biodegradability characteristics by enzymatic degradation with lysozyme solutions of varying concentrations. Completely water-soluble products were obtained within 48 h. *In vitro* cytotoxicity assays of the CM-hydrogel and its extract against three cell lines, NCTC clone 929, IMR-90 and MG-63, indicated the hydrogel was non-cytotoxic with cells able to adhere and proliferate well on the hydrogel.

## 1. Introduction

Chitin and its primary derivative, chitosan are biopolymers of immense interest for many environmental, industrial and medical applications. Chitosan is more popular as it is soluble in dilute acids permitting ready characterization and manipulation into gels, films and rods. Strong hydrogen bonding exists between chitin chains making chitin intractable, soluble only in harsh solvents such as concentrated acids that degrade chitin, limiting its widespread utility. More recently, the use of polar solvents such as dimethylacetamide has somewhat alleviated chitin’s intractability issue. Research in the chemical derivatization of chitin and chitosan has been a long held approach to render chitin and chitosan materials more easy to work with by generating soluble derivatives *via* functionalization of their pendant side groups. Additionally, these new derivatives extend the utility of chitin and chitosan by virtue of the derivative’s character incorporated with those of the base biopolymer. One such favorable outcome is that of generating hydrogels.

Natural and synthetic hydrogels present many significant advantages for potential biomedical application to the human body. They can: protect cells and fragile drugs (peptides, proteins, oligonucleotides, DNA) *in vivo*; transport nutrients to, and remove waste-products from cells; be modified with cell adhesion ligands; injected *in vivo* as a liquid that gels at body temperature; and most of them are non-toxic to body tissue [1]. Hydrogels made from biopolymers are more promising due to their intrinsic properties such as non-cytotoxicity and general biodegradability. Several biopolymers-derived hydrogels have been applied in tissue engineering. For example, gelatin gels have been utilized for delivery of growth factors to promote vascularization of engineered new tissue [2]. Dextran is a well-known polysaccharide that has been used as a plasma expander and drug carrier because of its good tissue compatibility. Its derivative, carboxyl-methyl-dextran is a pH-sensitive hydrogel for drug delivery [3,4,5].

Photo-crosslinking is a common and convenient method to prepare hydrogels without the use toxic crosslinking agents. Synthetic polymers or derivatives of biopolymers containing unsaturated functional groups have been utilized as photo-crosslinkable precursors for making hydrogels. Photo-crosslinked hydrogels have been investigated extensively for a number of biomedical applications including the prevention of thrombosis and post-operative adhesion because of the ability to introduce the biomaterials with minimal invasiveness as injectables that solidy *in situ* [6]. Smeds *et al.* developed methacylated-alginate and methacylated-hyaluronan as *in situ* hydrogels with favorable rheological and swelling properties [7]. Vyavahare *et al.* reported four different types of photo-crosslinked hydrogels varying in acrylate types derived from the copolymer of poly(ethylene glycol) and lysine as backbone [8].

Functionalization of chitin and chitosan with photo-crosslinkable groups is known. Tanodekaew *et al.* have prepared acrylate-grafted chitin by the reaction of chitin with acrylic acid in the presence of strong sulfuric acid under heterogeneous conditions. The acrylate cross-linkable functional group on chitin was used to produce a chitin-based hydrogel that can be used as temporary skin substitutes for wound dressing application [9]. Separately, Ishihara *et al.* developed a photo-crosslinkable chitosan derivative by introducing azide functional groups onto the backbone of chitosan. The solution of the chitosan derivative formed a hydrogel when irradiated with UV [10]. These illustrations augurs well that cross-linkable functional groups present a channel for realizing chitin and chitosan hydrogels.

In this study, a novel photocrosslinkable-cum-water-soluble chitin derivative was prepared by the action of methacrylic acid on low molecular weight chitin under mild conditions to introduce UV-active groups onto the chitin backbone. The corresponding hydrogel was readily prepared by the UV-irradiation of chitin-methacrylate aqueous solution without the use of any other crosslinking agents or synthetic monomers. Surface morphology and enzymatic degradation of the chitin-methacrylate hydrogel were also investigated in this preliminary work.

## 2. Results and Discussion

Chemical derivatization of chitin and chitosan have used starting materials of varying molecular weights under heterogeneous as well as homogeneous reaction conditions. As a rule higher molecular weights and heterogeneous reactions tend to give products that are diverse in properties and difficult to characterize. Lower molecular weights and homogeneous reactions give more consistent products as the biopolymer nature permits. In this work, we have elected to utilize shrimp shells as the source of chitin from a reliable supplier. Shrimp shell chitin has typical molecular weights <500 KDa and a lower inorganic content compared to crab or lobster shells. Therefore, the isolation of chitin is normally milder and a better quality raw material is expected.

### 2.1. Preparation and Characterization of Low Molecular Weight Chitin

The solubility of chitin in DMAc/LiCl improves dramatically with decreasing molecular weight that greatly facilitates chemical modifications compared to high molecular weight chitin solutions that have high viscosities that do not readily enable chemical modifications [11]. Chitin is readily depolymerized to lower molecular weight chitin, oligmers or a monomer mixture of glucosamine and N-acetyl-glucosamine by either enzymatic or mineral acids hydrolysis. Hydrochloric acid has been shown effective in hydrolyzing chitin in a short time without significant deacetylation [12]. In addition, there are no side reactions with HCl, compared to H_2_SO_4_ where O-sulfation has been found [13].

**Figure 1:**
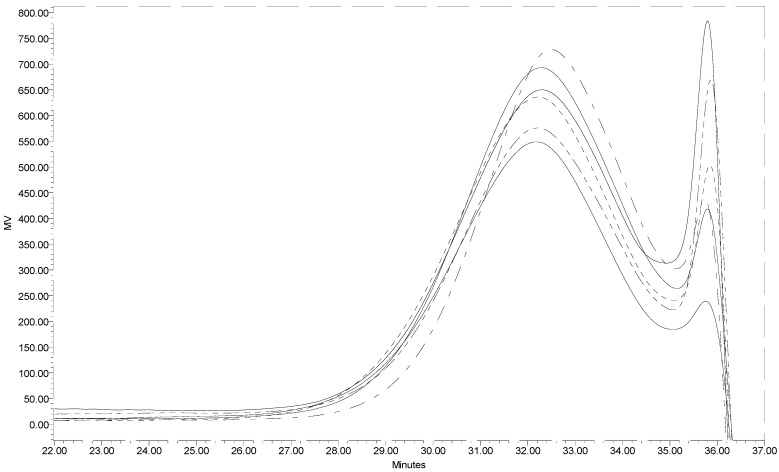
Segments of the GPC profiles of six low molecular weight chitin samples.

Figure 1 presents the GPC profiles of six individual batches of low molecular weight chitin prepared under the same hydrolysis conditions. The closeness of the GPC profiles for the six samples warrants that reproducibility is acceptable. The weight average molecular weight for the low molecular weight chitin prepared in this work based on an average of the six samples was determined to be 10KDa. This means that concentrated HCl was able to depolymerize high molecular weight chitin (in this work estimated to be ~240 KDa) in a short time (15 min). Utilization of a 12 KDa mw cut-off dialysis membrane in this instance was permissible because chitin even at 10KDa is water insoluble and is retained in the dialysis membrane. The dialysis membrane permits the separation of water-soluble small molecular weight oligomers considered as by-products of chitin hydrolysis, giving a narrower molecular weight distribution.

The degree of N-acetylation was estimated from FTIR to be 88% and is high enough to satisfy the hydrolysis product as chitin. The average yield of low molecular weight chitin from the acid hydrolysis was approximately 60%, suggesting that the acid hydrolyzed the remainder component to even smaller oligomers of N-acetylglucosamine and N-glucosamine.

### 2.2. Preparation and Characterization of Chitin-Methacrylate

**Figure 2:**
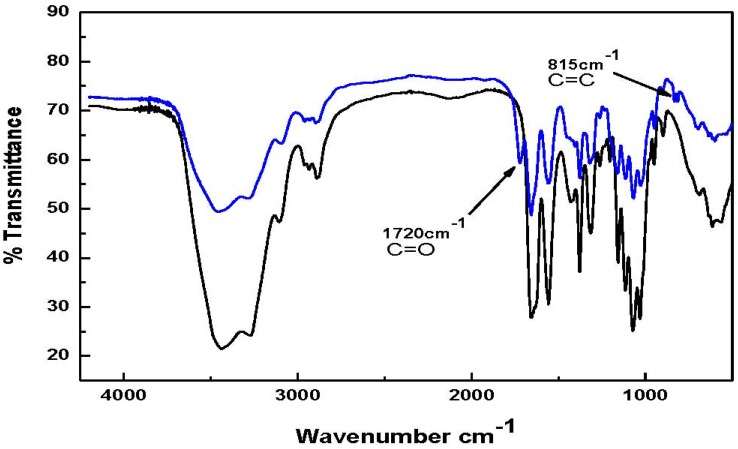
FT-IR spectra of low molecular weight chitin and chitin-methacrylate Top spectrum: Chitin-Methacrylate (CM); Bottom spectrum: Low molecular weight chitin.

**Figure 3:**
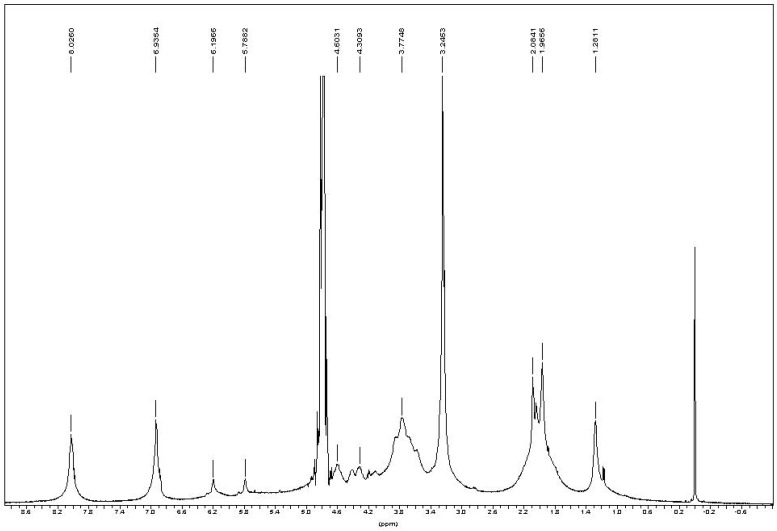
^1^H-NMR of chitin-methacrylate in D_2_O.

**Figure 4:**
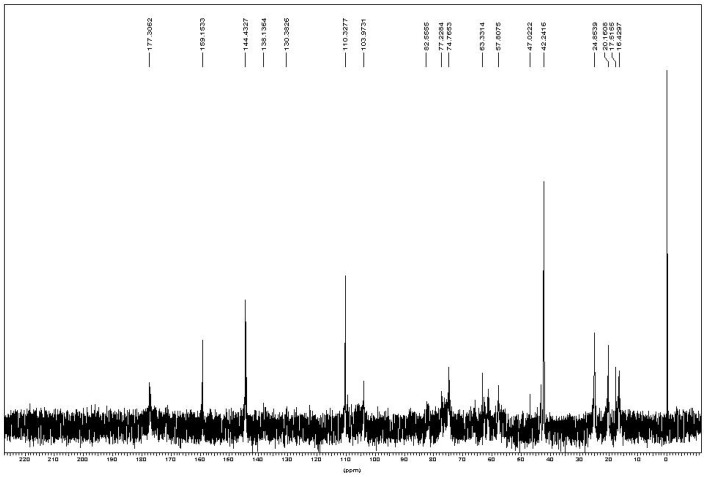
^13^C-NMR of chitin-methacrylate in D_2_O.

The chemical derivatization of chitin with methacrylic acid, in the presence of N,N'-dicyclocarbodiimide (DCC) as coupling agent and 4-dimethylaminopyridine (DMAP) as co-catalyst, to give chitin-methacrylate was straightforward. The FT-IR and NMR spectra shown in Figure 2, Figure 3, and Figure 4 were used to elucidate the structure. In Figure 2, the FT-IR spectrum for chitin-methacrylate show the additional band at 1720 cm^−1^ attributed to the stretching of the methacrylate carbonyl group together with another band at 815 cm^−1^ ascribed to the pendant vinyl group of methacrylate on the chitin backbone [14]. Both absorbances are absent in the low molecular weight chitin spectrum.

In the ^1^H-NMR spectrum of chitin-methacrylate (Figure 3), there are two peaks at 5.18 and 5.77 ppm corresponding to the two the protons of –C=CH_2_ of the methacrylate group, while the 1.96 ppm peak is attributed to the -CH_**3**_ group on methacrylate. Other chemical shifts are assigned to the chitin backbone protons, *i.e.*, 4.6 ppm at C1, the broad peaks between 3.4–4.2 ppm are the C2–C5, and the typical chemical shift at 2.08 ppm to the -CH_3_ on the N-acetyl group of chitin.

**Scheme 1:**
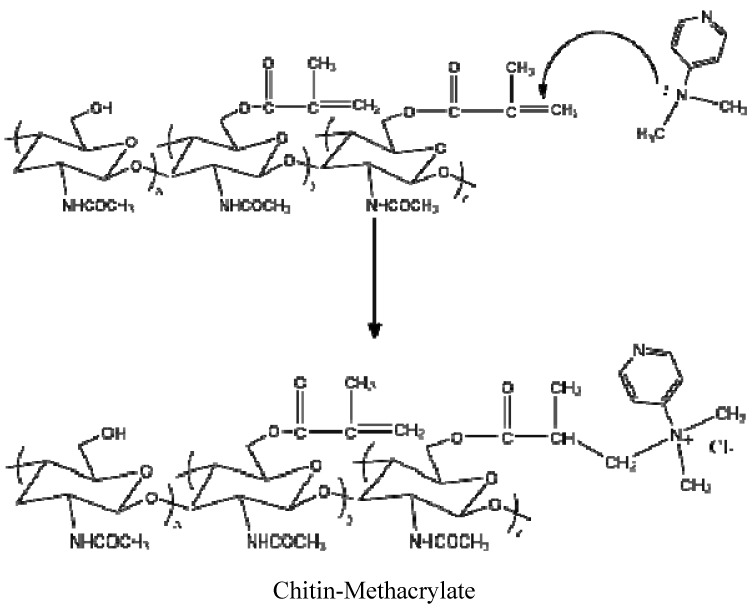
Reaction of methacrylate group with 4-dimethylaminopyridine (DMAP).

There were three additional peaks located at 8.21, 6.93 and 3.24 ppm assigned to DMAP [15]. Persistent washing of chitin-methacrylate (CM) with base, acid and various other solvent combinations did not remove the DMAP contributions in the NMR spectrum and it was concluded that the DMAP moiety was covalently bonded to the chitin backbone. This presence of DMAP on the chitin backbone was rationalized in terms of the occurrence of a further reaction between the grafted methacrylate groups in the presence of excess DMAP as shown in Scheme 1. The result is the presence of a cation centered on the DMAP base rendering CM water-soluble. The existence of the double bond suggests that the complete CM structure is a mixture of pure methacrylate and methacrylate-dimethylamino complex grafted onto the chitin backbone.

In the ^13^C-NMR spectrum (Figure 4), two weak peaks at 130.3 and 138.1 ppm were assigned to the two carbons at the unsaturated (–C(CH_3_)=CH_2_) of methacrylate group. There were two peaks near 20 ppm assigned to the methyl group on chitin’s N-acetyl group (24 ppm, –NHCOCH_3_) and methacrylate group (20 ppm, –C(CH_3_)=CH_2_). Another two peaks near 16 ppm were probably due to the carbons on the methacrylate group in the additional reaction at 17.5 ppm and 16.5 ppm. The obvious strong chemical shifts at 42.2, 110.3, 144.4 and 159.1 ppm were assigned to DMAP. Other chemical shifts corresponded to the chitin backbone found in the spectrum and summarized as Table 1.

**Table 1:** Chemical shifts of carbon belonging to the chitin backbone structure.

	C1	C2	C3	C4	C5	C6	NHCOCH_3_
^13^C chemical shifts	103.9	57.8	74.7	82.5	77.2	63.6	177.3

From the characterization of CM, we conclude that the attachment of the methacrylate group onto chitin was achieved with a final chemical structure depicted in Scheme 1. Unfortunately, the degree of substitution of the methacrylate group on the chitin backbone could not be obtained from ^1^H-NMR by comparing the integration of protons on –C=CH_2_ to –CH_3_ on chitin. This was because the chemical shifts on either chitin’s repeat unit or the methyl group of the N-acetyl group could not be clearly distinguished in spite of the distinct chemical shifts of the unsaturated group were distinct.

CM was found to be soluble in polar organic solvents and water. For DMSO and DMAC (containing 5% LiCl w/v), CM was soluble up to about 20% (w/v) in both solvents. In pure water, CM dissolved to a maximum concentration of around 7% (w/v), which was a very viscous solution. 

### 2.3. Thermogravimetric Analysis (TGA) Profile of CM

**Figure 5:**
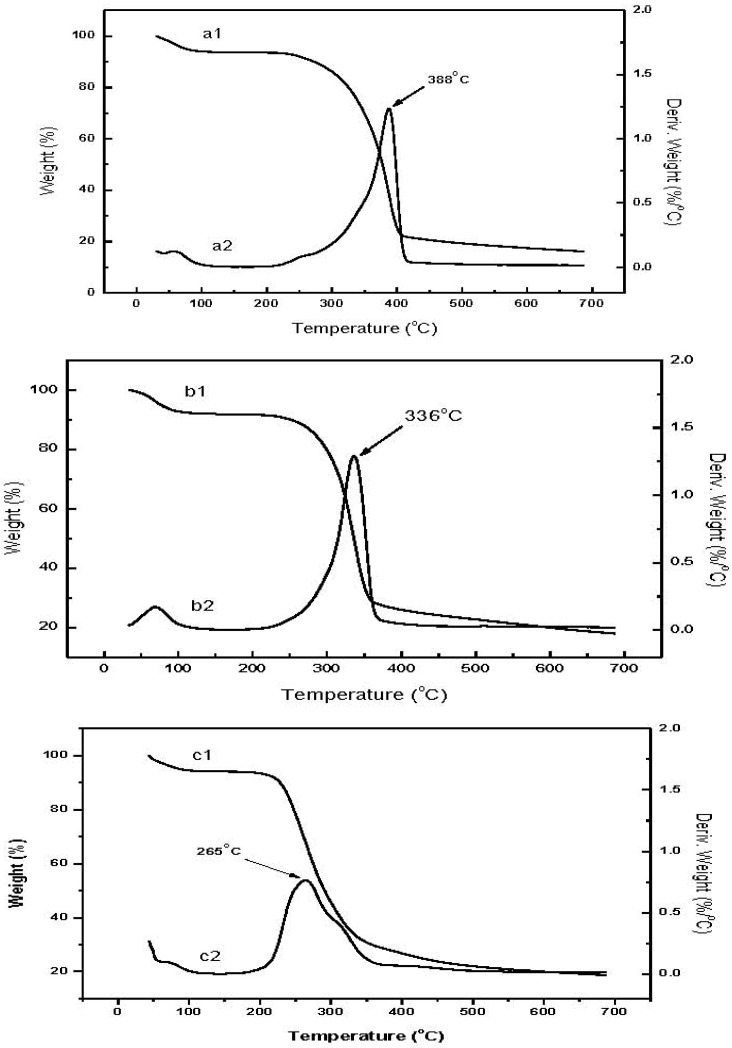
Thermograms of raw chitin, low molecular weight chitin and CM: a1, b1, c1, correspond to the weight loss curve; a2, b2, c2, correspond to the derivative weight loss curve.

Figure 5 shows the TGA profiles of raw chitin, low molecular weight chitin, and CM. Weight loss curves for the raw chitin (a1) and low molecular weight chitin (b1) have two decomposition stages: the first, around 50–100 °C, is attributed to the loss of residual water; the second stage thermal decomposition of the biopolymer, is a little different for the two chitin thermograms, 240–410 °C for raw chitin and 220–370 °C for low molecular weight chitin. The lower onset of decomposition for the low molecular weight chitin is probably a consequence of the lower degree of polymerization [16]. From the derivative curves, the temperature of maximum weight loss (T_max_) is 388 °C for raw chitin (a2), higher than that of low molecular weight chitin at 336 °C (b2).

The decomposition profile for CM (c1) appears more elaborate compared to the chitin profiles. The first stage from 50–100 °C is again attributed to the loss of residual water. The primary stage spans from 190–380 °C. The derivative curve profile (c2) suggests the existence of 2 overlapping components, the lower probably contributed by the less stable methacrylate component while the higher is attributed to the stabler methacrylate-dimethylaminopyrine complex component. Based on the derivative curves, we conclude that the ratio of methacrylate to dimethylaminopyrine complex is approximately equal. These results are similar to the acrylate-chitin consistent with accepted findings that chemical modification results in lower thermal stability of the chitin derivative compared to the starting chitin material [9,16].

### 2.4. Crystalline Character of CM by XRD

**Figure 6:**
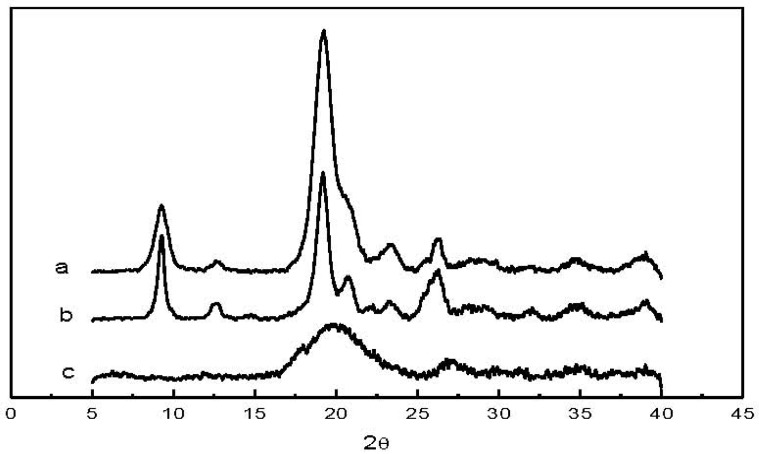
X-ray diffraction patterns of (**a**) raw chitin; (**b**) low molecular weight chitin; and (**c**) CM.

The XRD patterns of raw chitin, low molecular weight chitin and CM are presented in Figure 6. Five main peaks could be observed in both the raw chitin (a) and low molecular weight chitin (b) patterns at 2θ = 9.27, 12.66, 19.19, 23.20, and 26.31°, in agreement with reports from the literature [17]. The results indicate that chitin’s crystalline structure remained essentially unchanged after acid hydrolysis. However, almost no crystal structure was found in the CM XRD pattern (c). This indicates that the chemical modification of chitin to CM resulted in an amorphous structure.

### 2.5. Preparation and Characterization of CM-Hydrogel

**Figure 7:**
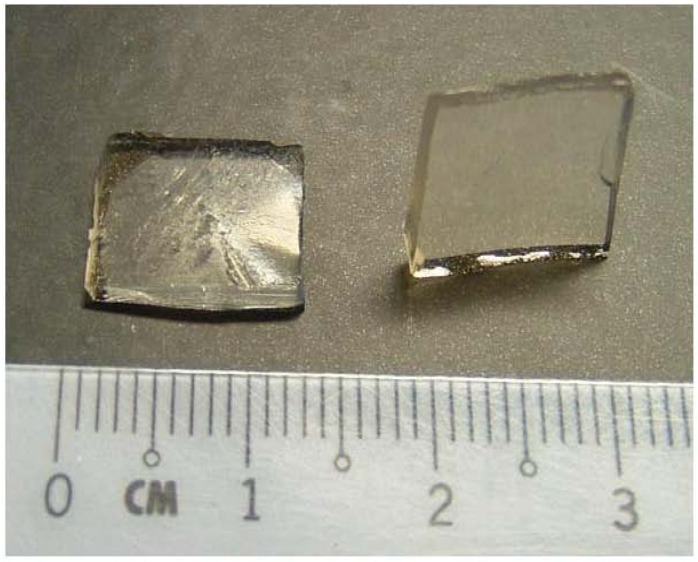
Photograph of CM-Hydrogel after irradiation for 1 hour. Left: Cross sectional view; right: Hydrogel standing on one of its edge.

One of the more popular potential biomedical applications of chitin and chitosan materials would be as hydrogels [1]. The existence of intact double or vinyl bonds on the methacrylate group grafted onto the chitin backbone opens the possibility to UV or heat initiated cross-linking of CM to generate a hydrogel. In this work, CM-hydrogel was prepared from its aqueous solution upon UV-irradiation in the presence of a photo-initiator. The CM aqueous solution converts to a transparent hydrogel in about 10–15 min as indicated by a clear separation of a gel from the liquid. Figure 7 shows a photograph of a CM hydrogel obtained by irradiation for 1 h. The gel was firm to touch as well as able to stand when placed on its edge. In the absence of the photo-initiator no hydrogel was formed even after several hours of UV-irradiation.

### 2.6. *In Vitro* Lysozymal Degradation of CM-Hydrogel *in Vitro* by Lysozyme

**Figure 8:**
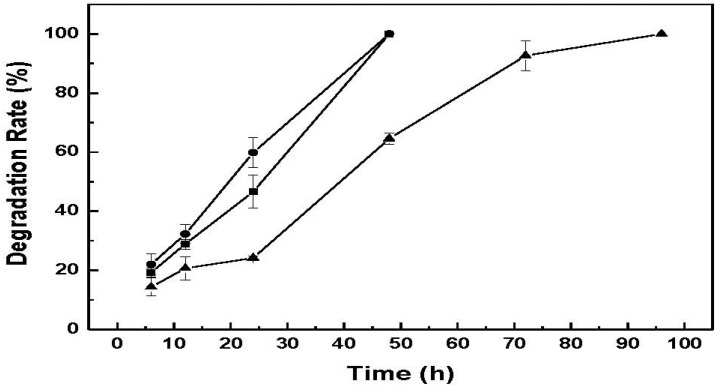
Enzymatic degradation of CM-hydrogel in different concentrations of lysozyme solutions. ―●―: 4 mg/mL, ―■―: 2 mg/mL, ―▲―: 1 mg/mL.

Chitin and its derivatives in film or gel forms readily hydrolyze in the presence of lysozyme [18]. As chitin-based hydrogels have potential in biomedical applications, one fundamental aspect to ascertain is hydrogel biodegradability susceptibility. The lysozyme degradation of CM-hydrogel was carried out with different lysozyme concentrations in 0.01 M pH 7.0 PBS at 1.0 mg/mL, 2.0 mg/mL, and 4.0 mg/mL in 37 °C water bath. Figure 8 shows the degradation rate of CM-hydrogel using these lysozyme concentrations. The results indicate that the photo-crosslinked CM-hydrogel can be totally degraded within 48 h by 2 mg/mL or 4 mg/mL of lysozyme solution, while a longer time of 96 h was required for the 1 mg/mL lysozyme solution for the complete dissolution of hydrogel. Therefore the amount of enzyme present is critical for the steady degradation of hydrogel. In addition, the effective enzymatic degradation by lysozyme indicates that the contiguous N-acetyl groups on chitin that serves as the active site of lysozyme still exist in the low molecular weight chitin-methacrylate after acid hydrolysis and chemical modification [15]. While it is noted that the utility of a hydrogel that degrades within 48h is nomimal, the susceptibility to biodegrade in the presence of enzymes is demonstrated. In perceived use, the amount of enzymes would be expected to be much less and therefore a longer “*in situ*” lifetime would be expected. Furthermore, CM can be combined with other photo-crosslinkable materials that would further alter the degradation profile. This ability to biodegrade augurs well for CM-hydrogel, and in general, chitin-methacrylate, for biomedical applications.

### 2.7. *In Vitro* Cytotoxicity

**Figure 9:**
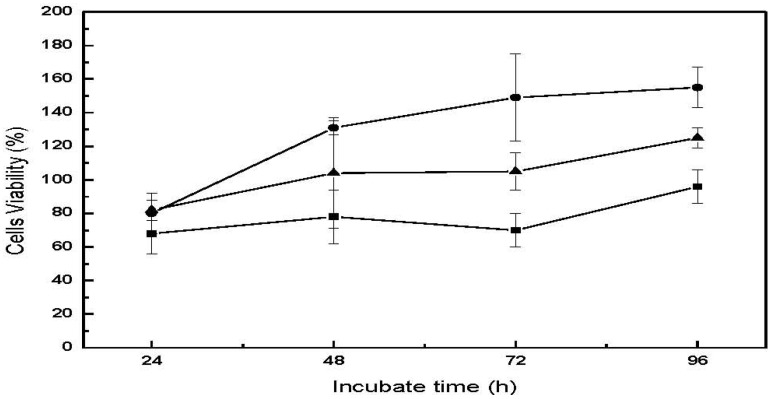
Cell viability as a function of incubation time for the direct contact cytotoxicity assay of CM-hydrogel. ―●―: CCL-186, ―▲―: CRL-1427, ―■―: CCL-1.

The *in vitro* cytotoxicity evaluation of biomedical materials at the research stage is a good tool for identifying potential safety issues of newly prepared materials as it utilizes only cell culture and is relatively fast and straightforward to perform. The results give useful indications of the new material’s suitability for further product development. Three types of cell lines were used for assessing the cytotoxicity of CM-hydrogel directly or indirectly using its extract in culture medium. The percent cells viability is shown in Figure 9 and Table 2. Cell viability for all three cell lines following direct contact with the CM-hydrogel were approximately 70% at 24 h, and increasing to 150% for human fibroblast (CCL-186), 125% for osteoblast (CRL-142) and 96% for mouse fibroblast (CCL-1) after 96 h incubation. In particular, the cell viability for the two human cell lines were found to be above 100% after 48 h, probably because the larger size of the human fibroblasts and osteoblasts compared to the mouse fibroblasts facilitated their easier attachment and proliferation on the hydrogel surface. These values remained more or less constant up to 96 h. This result is similar to that reported by Chow *et al.* who also found that the cell viability of CCL-1 mouse fibroblast cells following incubation with chitin-fluorinated derivatives was 20% lower than that of CCL-186 human fibroblast cell line [19]. In addition, the CM-hydrogel would be expected to be uneven compared to polystyrene, a situation that will favor easier attachment and growth of the human osteoblast and fibroblasts on the hydrogel leading to higher cell viability [20].

**Table 2:** Viability of CCL-1, CCL-186 and CRL-1427 cells following incubation for 24 and 48 h with the CM-hydrogel extract.

Incubation Time	Cells Viability (%) (Mean ± SD)		
	CCL-1	CCL-186	CRL-1427
24 h	78 ± 3	98 ± 7	77 ± 9
48 h	62 ± 2	98 ± 3	92 ± 8

Similarly, cells exposed to the undiluted hydrogel extract showed comparable viability profiles relative to control (Table 2). The two human cell lines exhibited viabilities of more than 90% compared to the control, while the mouse cell line, although lagging behind the human cell types, was 60% viable after 48 h of incubation with the extract. From the material safety aspect, therefore, the CM-hydrogel can be regarded as potentially non-toxic, holding out promise for biomedical applications such as wound healing, tissue engineering and orthopedic implants.

### 2.8. Morphology of Cells Cultured on CM-Hydrogel Surface

**Figure 10:**
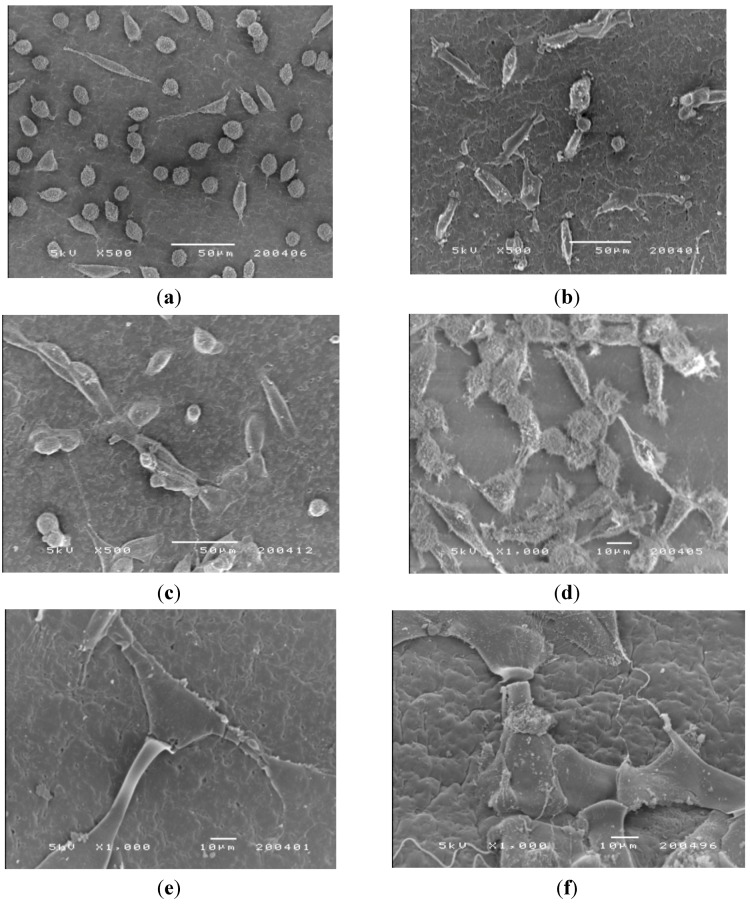
SEM photomicrographs of the morphology of CCL-1, CCL-186 and CRL-1427 cells cultured on the CM-hydrogel. Day 2: (**a**) CCL-1; (**b**) CCL-186; (**c**) CRL-1427. Day 5: (**d**) CCL-1; (**e**) CCL-186; (**f**) CRL-1427.

SEM was used to examine the adhesion, spread and proliferation of the three cell lines on the surface of CM-hydrogel after incubation at 37 °C for 2 and 5 days. Representative SEM micrographs (Figure 10a–c) taken on day 2 show unequivocal adhesion of all cell types on the surface of the CM-hydrogels. The mouse fibroblasts (CCL-1) were mainly round-shaped with a few cells exhibiting the spindle shape. In contrast, both the CCL-186 and CRL-1427 cells were spindle-shaped, and they were widely spread out on the surface of the CM-hydrogel. This observation supports the cytotoxicity data in showing that the mouse fibroblasts indeed grew much slower, and with delayed colonization, compared to the human fibroblasts and osteoblasts on the CM-hydrogel. The SEM micrographs of the mouse fibroblasts on day 2 was similar to that reported by Muzzarelli *et al.* that showed only a few spindle shaped mouse fibroblasts cells just starting to spread on a di-butyryl chitin film.

Photomicrographs of the cell-hydrogel surface on day 5 (Figure 10d–f) show extensive cell proliferation that spread across the material surface. The fibrils of the cells (white arrows) extended on the surfaces of the CM-hydrogel, showing evidence of cell-cell connection, indicating good cell adherence.

## 3. Experimental Section

### 3.1. Materials

Chitin was purchased from Eland Corporation Ltd, Thailand. Anhydrous DMAc was purchased from Sigma. Methacrylic acid, N,N'-dicyclocarbodiimide (DCC), methacrylic acid and 4-dimethylaminopyridine (DMAP) were purchased from Fluka. LiCl was obtained from J.T. Baker. Hydrochloric acid was purchased from Merck, Germany. The photo-initiator Irgacure 2959 was a generous gift from Ciba Specialty Chemicals, Singapore. Other solvents used were of reagent grade, used without further purification.

### 3.2. Preparation of Low Molecular Weight Chitin

4.0 g of purified chitin powder were placed in a 250 mL round-bottom flask (RBF) containing 120 mL of concentrated HCl solution (37%) and magnetic stirrer. The RBF was placed in a water-bath at 40 °C and stirred. After 10 min, the RBF was cooled in an ice-bath followed by pouring the reaction mixture into a 600 mL beaker. The hydrolyzed chitin was carefully neutralized with NaOH solution in an ice-bath, giving a white precipitate that concomitantly became a milky solution. The solution was centrifuged and dialyzed (*Sigma*-molecular cut-off of 12 KDa) with tap water for 2 days and subsequently lyophilized to give hydrolyzed low molecular weight chitin powder.

### 3.3. Preparation of Chitin-Methacrylate (CM)

**Scheme 2:**
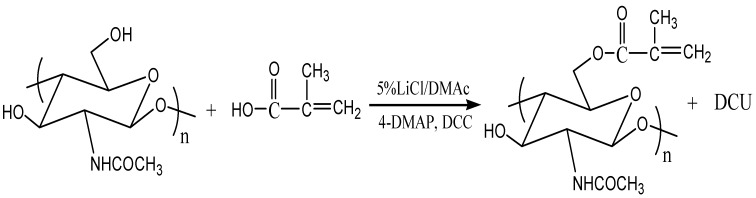
Preparation of chitin-methacrylate (CM).

The esterification of a carboxylic group with a hydroxyl group in the presence of N,N'-dicyclohexylcarbodiimide and 4-dimethylaminoamine is a traditional method that can be conducted under milder reaction conditions than the acylation of acyl-halide with alcohol or the esterification of carboxylic acid with alcohol in the presence of concentrated sulfuric acid. Scheme 2 shows the reaction used in this work for the esterification of chitin and methacrylic acid in 5% LiCl/DMAc solvent system.

In a 100 mL RBF, 0.8 g of the prepared low molecular weight chitin powder was dissolved in 20 mL of 0.5% LiCl/DMAc to make 4.0% (w/v) chitin solution. Using a syringe, 2.0 mL (0.0236 mol, 6:1 as molar ratio based on pyranose of chitin) of methacrylic acid was added slowly to the chitin solution. This was followed sequentially by the addition of 0.196 g (40% mol of molar of repeat unit of chitin) of 4-DMAP (dissolved in 4 mL of anhydrous DMAc prior to add) and 4.88 g of DCC (dissolved in 4 mL of anhydrous DMAc, same molar ratio as in methacrylic acid). The reaction mixture was stirred for 48 h at room temperature. The resultant white precipitate DCU (dicyclohexylurea, the by-product of DCC) was removed by filtration and acetone added to the reaction solution to precipitate the chitin-methacrylate (CM) product. The CM product was washed with acetone (3 × 50 mL) and dissolved in 50 mL of D.I. (deionized) water, dialyzed (*Spectropor*-molecular cut-off of 1 KDa) against D.I. water at 4 °C for 5 days to remove water-soluble small molecules and finally lyophilized to obtain fluffy chitin-methacrylate.

### 3.4. Preparation of CM-Hydrogel by UV-Irradiation

CM-hydrogel was prepared by dissolving the desired amount of CM and Irgacure 2959 (5% w/w based on CM) in D.I. water to give a homogenous precursor solution that was subsequently placed under a UV source (Vilber Lourmat, France) at 365 nm for 1 h. The solution formed a hydrogel in the first 10–15 min; the prolonged irradiation exposure time was to increase the crosslinking degree.

### 3.5. Determination of Molecular Weight Distribution of Hydrolyzed Chitin by GPC

The molecular weight distribution of low molecular weight chitin powder was obtained with a Waters Gel Permeation Chromatography system equipped with a Waters LC (model 515) pump together with a Waters 410 differential refractometer as the detector. Pullulan standard P-82 (Shodex, Japan) was used as the molecular weight reference. The low molecular weight chitin powder was dissolved in 5% LiCl/DMAc to make a 2%(w/v) chitin solution and eluted with 5%LiCl/DMAc at 0.8 mL/min through a three column series (Phenomenex 10 Linear, 300 mm × 7.8 mm × 10μm) equipped with a guard column at 65 °C, the detector temperature was set at 40 °C.

### 3.6. FT-IR Characterization *[21]*

The degree of N-acetylation of low molecular weight chitin and the presence of the methacrylic group on chitin-methacrylate was evaluated by FT-IR. A small amount of low molecular weight chitin or chitin-methacrylate was mixed well with fine KBr powder, made into pellets and scanned on the FT-IR spectrometer (Bio-Rad Laboratories, Cambridge, MA, USA) for 32 times at 4 cm^−1^ resolution. The degree of N-acetylation was estimated using the equation below, where A_1320_ is the absorbance at 1320 cm^−1^ and A_1420_ the absorbance at 1420 cm^−1^.

D.A.: A_1320_/A_1420_ = 0.3822 + 0.03133DA [7]

### 3.7. Liquid Nuclear Magnetic Resonance (NMR)

^1^H-NMR and ^13^C-NMR were used to determine the chemical structure of chitin-methacrylate. Samples were dissolved in D_2_O and spectra recorded with a Bruker ACF 300 NMR (300MHz) instrument. The number of scan was 32 for ^1^H-NMR and overnight for ^13^C-NMR.

### 3.8. Thermo Gravimetric Analysis (TGA)

Thermogravimetric analysis was used to study the decomposition profiles of low molecular weight chitin powder and CM, and contrasted to the raw chitin obtained from shrimp shells. Approximately 10 mg of dried sample (oven for chitin; freeze-drying for CM) were placed in alumina pans, and heated from ambient temperature to 700 °C at 10 °C/min under nitrogen gas at a flow rate of 100 mL/min in the TGA 2960, TA Instruments, Inc. thermal analyzer. The data was analyzed using Universal Analysis Software (TA Instruments, Inc).

### 3.9. Powder X-Ray Diffraction (XRD)

Wide angle X-ray diffraction was utilized to determine the crystalline structure of chitin, low molecular weight chitin, and CM. The powder of each sample was pressed in the holder and run on an X-ray diffractometer (Siemens D5005) from 2θ = 5° to 40° at the scanning rate of 0.01°/second.

### 3.10. Enzymatic Degradation Study [22]

*In vitro* enzymatic degradation of CM-hydrogel was conducted with lysozyme concentrations of 1.0 mg/mL, 2.0 mg/mL, and 4.0 mg/mL in 0.01M pH7.0 PBS in a 37 °C water bath; various concentrations of lysozyme solution. The pre-weighed hydrogel was soaked in a beaker containing 2 mL of lysozyme solution at 37 °C for 6, 12, 24, 48, 64 and 96 h, removed when the specified time was reached, and with D.I. water to wash away residual lysozyme and air-dried until constant weight.

The degradation rate was expressed as:

Degradation Rate (%) = (W_x_ − W_d_)/W_x_

where W_x_ is the mass of starting xerogel, and W_d_ is the mass of dried sample after enzyme interaction. The experiments were performed at least in triplicates.

### 3.11. Cytotoxicity Evaluation of CM-Hydrogel

Cytotoxicity evaluation of CM-hydrogel was assessed using both the direct contact and extract methods according to ISO 10993-5 [23]. The CM-hydrogel surface or an extract of a CM-hydrogel sample were exposed to the cells for a pre-determined incubation period and the cells examined by MTT assay to quantify cytotoxicity. MTT assay is a rapid colorimetric method based on the cleavage of a yellow tetrazolium salt (3-{4,5-dimethylthiazol-2-yl}-2,5-diphenyl tetrazolium bromide) to form purple formazan crystals by mitochondrial enzymes of metabolically active cells. The formazan crystals are dissolved in DMSO for quantification at a wavelength of 590 nm [4].

#### 3.11.1. Cell Culture

Three cell lines, NCTC clone 929 (mouse fibroblast, ATCC CCL-1), IMR-90 (human lung fibroblast, ATCC CCL-186), and MG-63 (osteoblast from osteosarcoma, ATCC CRL-1427) were used to assess the cytotoxicity of CM-hydrogel *in vitro*, as well as the adhesion morphology of cells grown on the hydrogel surface. All cells were cultured in Minimal Essential Medium (MEM, Gibco BRL) containing 10% heat-inactivated fetal bovine serum (Gibco BRL), 0.1% non-essential amino acids (Sigma), 1.5 g/L sodium bicarbonate (Cell Culture Grade, US Biological), 0.1 g/L penicillin (Sigma) and 0.1 g/L streptomycin (Sigma) at 37 °C in a 5% CO_2_/95% H_2_O humidified atmosphere.

#### 3.11.2. Direct Contact Method

Pre-conditioning of CM-hydrogel was performed as follows. CM-hydrogel was dehydrated at room temperature for 48 h and placed in absolute ethanol for 3 h prior to sterilizing under a UV lamp overnight. The dehydrated CM-hydrogel was soaked in fresh culture medium for 24 h for the hydrogel to reach swelling equilibrium. The effects of UV irradiation on the hydrogel were not investigated. Sterility tests were not performed and acceptable sterility was concluded based on cell survivability and proliferation, and the lack of cloudiness in the culture media throughout the duration of the experiment (96 h).

Cells were seeded in the 24-well plates at 5 × 10^4^ cells per well and incubated for 24 h at 37 °C to allow cell adhesion. The medium was removed and the pre-conditioned hydrogel was gently placed in the wells, in contact with the cells. Polystyrene (negative control) and latex rubber (positive control) were also placed in the same manner. Fresh culture medium (1 mL) was added into each well and the 24-well plates incubated at 37 °C in 5% CO_2_ humidified atmosphere for 24 h, 48 h, 64 h, and 96 h. At each time interval, the culture medium in the wells of the designated 24-well plates was removed, and replaced with 200 μL of MTT solution (5 mg/mL in 1X PBS). Incubation was resumed for another 3 h to permit healthy cells to reduce the tetrazolium salt. Thereafter, the MTT solution was aspirated and 300 μL of DMSO was introduced to each well to dissolve the formazan crystals for quantification by colorimetric assay. The results were expressed as the percentage of viable cells. Each experiment was performed with at least three independent replicates.

The percent viable cells = (OD_S_ − OD_Pavg_)/(OD_Navg_ − OD_Pavg_) × 100%

where: OD_S_ is the optical density of sample; OD_Pavg_ is the average optical density of the positive control; OD_Navg_ is the average optical density of the negative control.

#### 3.11.3. Extract Method (Indirect Contact)

Fresh CM-hydrogels were cut to 1 cm (L) × 1 cm (W) × 1.5 mm (H), gently washed twice with Milli-Q water. The washings were combined to form the extract that was sterilized by passing through a 0.22 μm filter. Wells of 24-well plates were seeded at a lower amount of about 2 × 10^4^ cells/well and incubated for 24 h. The spent medium was replaced with 1mL each of the hydrogel extract and 1mL of culture medium and incubated for 24 h and 48 h. Fresh culture medium and 0.5% phenol solution were employed as negative and positive control, respectively. The positive control experiment was conducted in a separate plate to prevent phenol vapor from contaminating the hydrogel extract or the negative control. After the specified incubation time, the cells were subject to the MTT assay, the same procedure as employed in the direct contact method. Results are expressed as the mean percentage of viable cells of at least three replicates.

#### 3.11.4. Morphology of Cells Cultured on CM-Hydrogel Surface [24]

Scanning electronic microscopy (SEM) was used to observe the morphology and adhesion of cells cultured on the CM-hydrogel. 50 μL of cell suspension (1 × 10^6^ cells/mL) was dropped carefully onto the surface of the culture medium equilibrated CM-hydrogel in 24-well plates and incubated at 37 °C in 5% CO_2_/95% humidified atmosphere for 2 h for cells to attach onto the hydrogel. Subsequently, another 1 mL of fresh medium was carefully added to the well to cover the hydrogel surface and the plates were placed back into the incubator. After incubating for 2 days or 5 days, the individual CM-hydrogel was removed, washed twice with PBS and fixed with 2.5% glutaraldehyde for 1 h. The cells-fixed CM-hydrogel was dehydrated by placing in solutions of ethanol of increasing concentrations of 50%, 70%, 80% and 95% for 5 min each, twice in 100% ethanol for 15min each, and finally dried by critical point drying. The dried sample was gold sputtered for 45 s at 20 mA using an auto fine coater (JEOL, JFC-1600, Japan). A scanning electron microscope (JEOL, JSM-5200) was used to observe the cells morphology and its interaction with CM-hydrogel.

## 4. Conclusions

Chitin-methacrylate was readily prepared under homogeneous conditions. The chitin-methacrylate derivative was found to be photo- and heat-sensitive, permitting potential cross-linking reactions with a variety of substrates to produce new chitin-based compounds. The chitin-methacrylate derivative exhibits good solubility in water and DMSO, and can readily form a hydrogel upon UV-irradiation. The CM-hydrogel is firm, readily degraded by lysozyme and is non-cytotoxic towards human and mouse fibroblasts. It appears to facilitate good cell adherence on its surface, showing good potential as a gel implant, tissue-engineering scaffold and biodegradable drug delivery system. The demonstrated reactivity of the vinyl group on the methacrylate moiety implies that chitin-methacrylate can be used in situations where on-site cross-linking *via* UV-irradiation or heat initiation is desired.
